# Pain evaluation during gynaecological surveillance in women with Lynch syndrome

**DOI:** 10.1007/s10689-016-9937-x

**Published:** 2016-10-27

**Authors:** Jorien Helder-Woolderink, Geertruida de Bock, Harry Hollema, Magda van Oven, Marian Mourits

**Affiliations:** 1 University Medical Center Groningen, Groningen, The Netherlands; 2 Martini Hospital, Groningen, The Netherlands

**Keywords:** Lynch syndrome, Endometrial sampling, Pain, Surveillance, Endometrial cancer, VAS score

## Abstract

To evaluate perceived pain during repetitive annual endometrial sampling at gynaecologic surveillance in asymptomatic women with Lynch syndrome (LS) over time and in addition to symptomatic women without LS, undergoing single endometrial sampling. In this prospective study, 52 women with LS or first degree relatives who underwent repetitive annual gynaecological surveillance including endometrial sampling of which 33 were evaluated twice or more and 50 symptomatic women without LS who had single endometrial sampling, were included. Pain intensity was registered with VAS scores. Differences in pain intensities between subsequent visits (in LS) and between the two groups were evaluated. The use of painkillers before endometrial sampling was registered. If women with LS decided for preventive surgery, the reason was recorded. The LS group reported a median VAS score of 5.0 (range 0–10) at the first surveillance (n = 52) and at the second visit (n = 24). Women who repeatedly underwent endometrial sampling more often used painkillers for this procedure. During the study period 7/52 (13 %) women with LS choose for preventive surgery, another 4/52 (8 %) refused further endometrial sampling. Painful endometrial sampling was mentioned as main reason to quit screening. The median VAS score of the 50 symptomatic women was 5.0 (range 1–9). Endometrial sampling, irrespective of indication, is a painful procedure, with a median VAS score of 5.0. During subsequent procedures in women with LS, the median pain score does not aggravate although one in five women chose an alternative for endometrial sampling.

## Introduction

Women with Lynch syndrome (LS) have a significantly increased risk of endometrial cancer (25–70 %) depending on the type of gene mutation [1–6]. Currently there is evidence to support the efficacy of surveillance for the early detection of endometrial cancer in women with LS [1, 7–9]. The main goal of surveillance for endometrial cancer in LS is the detection of endometrial cancer at an early or premalignant stage and to provide early treatment with improved prognosis [10]. Data about effectiveness of annual gynaecological surveillance in women with LS or first-degree relatives at 50 % risk of LS who underwent surveillance in our hospital between 1991–2002 [11] and 2003–2012 [12] have been published before. Both studies concluded that surveillance with endometrial sampling is effective in the detection of (pre)malignant endometrial lesions in women with LS.

However, since gynaecological surveillance by transvaginal ultrasonography was extended by standard endometrial sampling, the clinical impression was that women more often complained about the painfulness of surveillance, that this deteriorated over time, and that this was associated with fear for the procedure and even opting out. Besides, the clinical impression was that LS women who came for annual surveillance more often reported intense pain during endometrial sampling than symptomatic women who had single endometrial sampling for bleeding problems (personal observation). For that we hypothesised that pain increases during subsequent endometrial samplings in women with LS.

The aim of this study was to evaluate the intensity and impact of pain during repetitive annual gynaecologic surveillance in women with LS or first-degree relatives at 50 % risk of LS. Pain scores of LS women were evaluated over time, and it was analysed if fear for endometrial sampling was a motivator to decide for preventive surgery or to stop the surveillance visits in women with LS. In addition, the pain scores were compared to pain scores of symptomatic women without LS who underwent single endometrial sampling for diagnostic reasons.

## Methods

Between January 2011–December 2015 women with LS (carrier of a pathogenic mutation in either *MLH1,MSH2, MSH6, Epcam* or *PMS2*) or first-degree relatives at 50 % risk of carrying a LS mutation were seen in the Family Cancer Clinic of the University Medical Center and a regional hospital (Martini Hospital) in Groningen, The Netherlands. All first degree relatives at 50 % risk of carrying a gene mutation were offered genetic testing and those (nine) women not willing to undergo that (yet), were also included in this study. All women were offered annual gynaecological surveillance by transvaginal ultrasound, endometrial sampling (Pipelle^®^) with measurement of the VAS score and serum CA125 measurement according to the Dutch National Guideline [13]. Data about endometrial samplings of some of these women have been described earlier in a study about the additional value of endometrial surveillance in women with LS [12].

The symptomatic non-Lynch women were seen in the outpatient clinic of the regional hospital in Groningen between January 2014 and August 2014. They underwent single endometrial sampling for diagnostic reasons. If women used daily painkillers they were excluded from the study. Written informed consent was obtained from all patients in this study. The ethics committee of the Martini Hospital in Groningen approved the study. All relevant data were entered into a separate password protected database, and protection of a patient’s identity was guaranteed by assigning study specific unique patient numbers.

### Measurements of outcomes

Pain measurement during endometrial sampling was evaluated at the surveillance visit with the Visual Analogue Scale (VAS score, range 0–10). Before endometrial sampling was performed, all women received information about the VAS score. They were instructed to give the pain score by using a VAS measuring staff (0 is no pain, 10 is the most severe pain you can imagine) directly after the endometrial sampling. At every surveillance visit it was documented if women with LS used painkillers before the endometrial sampling or if they declined further surveillance, and if so, for what reason.

### Data collection

For each woman, patient characteristics and clinical data including the medical history, use of daily (pain) medication, nulli- or multiparity, the age at the first surveillance, number of previous endometrial samplings, menopausal status, symptoms, results of TVU’s and of the endometrial sampling, pathology reports, CA 125 levels, pain measurement by VAS scores, treatment after endometrial abnormalities, decision for preventive surgery and the motivation for preventive surgery were collected.

#### Power analysis

To analyse a VAS score difference of 1 point between annual surveillance in the Lynch group and single surveillance in the symptomatic group, with a standard deviation of 2.0, alfa of 0.05 and a power of 80 %, two groups of 49 women each were needed.

### Data analysis

Characteristics of women with LS or first-degree relatives at 50 % risk and of the symptomatic group and their disease were described. The LS group and in addition to symptomatic women without LS were analysed on the outcome parameters: VAS scores, the use of painkillers before endometrial sampling, and in the LS group the decision to decline further surveillance. Differences between VAS scores over time were analysed for those women with LS or first-degree relatives at 50 % risk, having two visits. Then potential determinants (menopausal status, nulli/multiparity, and having a history with surveillance) were evaluated on our outcomes. This was done by Mann–Whitney U testing (in case of VAS scores) or Chi square testing (in case of use of painkillers and decision for preventive surgery). If women decided for preventive surgery, the reason was described. Data analysis was performed with SPSS statistics version 20.

## Results

In the women with LS group (n = 52), 97 annual gynaecological surveillances by TVU and endometrial sampling with VAS scores were performed. The mean age of the women at the first visit in this study period is 45 (range 33–69) years. A total of 33/52 LS women underwent subsequent endometrial surveillance after 1 year and in 24/33 endometrial samplings were performed (Table 1). The symptomatic group (n = 50) had a mean age of 59 (range 40–82) years (Table 1). In this latter group the indication for endometrial sampling was postmenopausal bleeding (n = 33; 66 %), irregular vaginal bleeding (n = 6; 12 %), menorrhagia (n = 7, 14 %) and other indications (n = 4; 8 %).

**Table 1:** Characteristics of patients and endometrial sampling

	Women with LS or first-degree relatives at 50% risk of the LS mutation					Symptomatic women
	First surveillance LS* (N = 52)	Second surveillance LS* (N = 33)	Third surveillance LS* (N = 17)	Fourth surveillance LS* (N = 5)	Fifth surveillance LS* (N = 3)	First visit (N = 50)
Mean age (range)	45.1 (33–69)	46.4 (34–70)	46.2 (35–71)	50.4 (40–66)	53.2 (41–67)	59.4 (40–82)
Menopausal status						
Premenopausal	40 (77 %)	26 (79 %)	13 (76 %)	3 (60 %)	2 (67 %)	12 (24 %)
Postmenopausal	12 (23 %)	7 (21 %)	4 (24 %)	2 (40 %)	1 (33 %)	38 (76 %)
Number of children						
Nulliparous	11 (21 %)	8 (24 %)	4 (24 %)	1 (20 %)	1 (33 %)	2 (4 %)
Primi/multiparous	36 (69 %)	21 (64 %)	11 (65 %)	3 (60 %)	1 (33 %)	33 (66 %)
Unknown	5 (10 %)	4 (12 %)	2 (11 %)	1 (20 %)	1 (33 %)	15 (30 %)
Started surveillance with endometrial sampling before the study period	28 (54 %)	21 (64 %)	13 (76 %)	5 (100 %)	3 (100 %)	NA

* First surveillance with endometrial sampling and VAS score, 28 (54%) women have had more surveillance visits with endometrial sampling before this study period

**Fig. 1:**
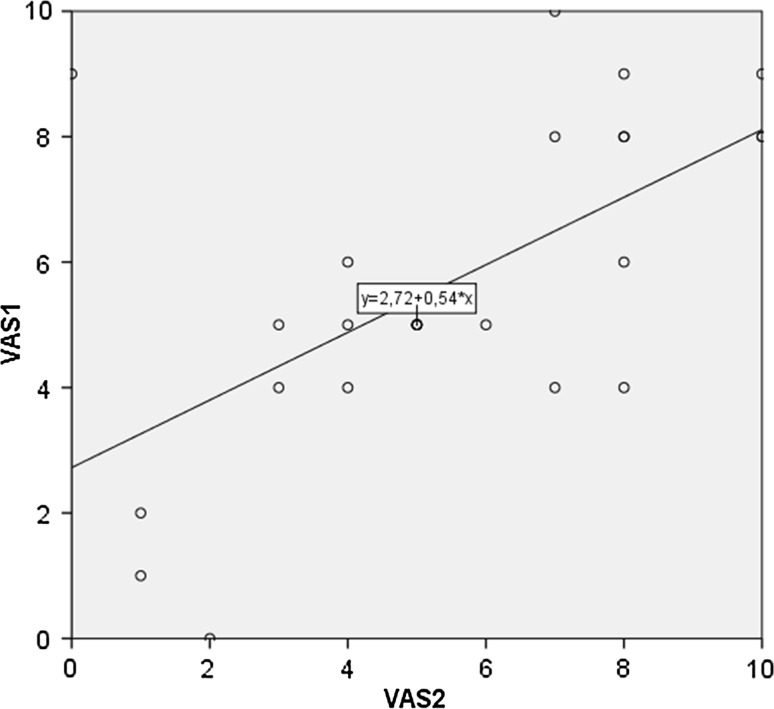
Level of VAS scores of the first and second visit among women with LS or first degree relatives (n = 24)

In the LS group, the median VAS score of the endometrial sampling at the first visit in this study period was 5.0 (range 0–10), see Table 2. The median VAS score at the second visit was also 5.0 (range 0–10). No women in the LS group used daily painkillers, 11/52 used painkillers (NSAID’s) 1–2 h before the endometrial sampling due to severe pain at previous visits. In the LS group, postmenopausal women (n = 12) reported a median VAS score of 6.5 (range 3–10) compared to premenopausal women (n = 40) who reported a VAS score of 5.0 (range 0–10) at the first surveillance visit (Table 3). In 11/52 nulliparous women, the median VAS score was 6.0 (range 2–9), compared to 5.0 (range 1–10) in 36 multiparous women. Women who started surveillance before 2011 reported the same median VAS score of 5.0 than women who started surveillance between 2011 and 2015, although they more often used painkillers (Table 3). In Fig. 1 is shown that most women report a substantial VAS score, which was highly individual with a wide range (0–10). The first and the second VAS score of the same women were comparable.

**Table 2:** Outcomes of VAS scores of endometrial sampling in women with LS and symptomatic women

	Women with LS or first-degree relatives at 50 % risk of the LS mutation					Symptomatic group
	First surveillance LS* (N = 52)	Second surveillance LS* (N = 33)	Third surveillance LS* (N = 17)	Fourth surveillance LS* (N = 5)	Fifth surveillance LS* (N = 3)	First visit symptomatic group (N = 50)
Median VAS score (range)	5.0 (0–10)	5.0 (0–10)	6.0 (1–9)	7.0 (4–7)	7.0 (1–9)	5.0 (1–9)
Used painkillers before endometrial sampling	11 (21 %)	9 (38 %)	5 (38 %)	4 (80 %)	2 (67 %)	0
Endometrial sampling	52 (100 %)	24 (73 %)	13 (76 %)	5 (100 %)	3 (100 %)	50 (100 %)
No endometrial sampling						
Only TVE during surveillance	0	4	0	0	0	0
Decided for preventive surgery	0	5	4	0	0	0

**Table 3:** Predictors for pain for women in the LS group

	Median VAS score at first visit (range)	*P* value	Used painkillers before first surveillance visit during study period (n = 11/52) (21 %)	*P* value	Decision for preventive surgery (n = 9) (17 %)	*P* value
Menopausal status						
Premenopausal (n = 40)	4.0 (0–10)	0.78*	8/40 (20 %)	0.14*	9/40 (23 %)	0.07*
Postmenopausal (n = 12)	6.5 (3–10)		3/12 (25 %)		0/12	
Number of children						
Nulliparous (n = 11)	6.0 (2–9)	0.39*	4/11 (36 %)	0.16*	1/11 (9 %)	0.34*
Primi/multiparous (n = 36)	4.0 (1–10)		5/36 (4 %)		8/36 (22 %)	
Unknown (n = 5)	8.0 (3–10)		2/5 (40 %)		0/5	
Start surveillance						
Before 2011 (n = 28)	5.0 (0–10)	0.97*	10/28 (36 %)	0.04	4/28 (14 %)	0.62*
After 2011 (n = 24)	5.0 (2–10)		1/24 (4 %)		5/24 (21 %)	

* Non significant

In the LS group five women stopped surveillance at the second surveillance visit and choose for preventive surgery and four women decided for preventive surgery at the third surveillance visit in this study period. In total 9/52 women (age 39–48 years, mean 44 years) underwent preventive surgery and 7/9 of these women reported pain during the endometrial sampling, besides fear for cancer, as the main reason to decide for preventive surgery. These nine women were informed about the option to get preventive surgery or to continue annual surveillance with a good counselling about the risks and the benefits of both procedures. All nine women who choose for preventive surgery had endometrial sampling before the operation with a median time between the endometrial sampling and the preventive surgery of 10 months (range 3–24 months). None of the women reported complaints of irregular blood loss. Four (8 %) women in the LS group refused one or more endometrial samplings because of fear for pain. One woman denied further surveillance after two painful endometrial sampling procedures (VAS 9 and 10).

Of nine women who choose for preventive surgery, the pathology report of endometrial tissue was normal in all women. The pathology report of 7/9 (78 %) women showed normal ovaries, one benign ovarian cyst was found and one patient was diagnosed with an unexpected FIGO stage IA, grade 1 intestinal ovarian adenocarcinoma within a teratoma. In this patient, at the last surveillance visit 3 months prior to the operation, a unilocular mass at the right ovary of 8.5 × 6 cm was seen with a normal aspect of the other ovary and absence of ascites. The level of CA 125 at that moment was 34 kU/L (Risk of Malignancy Index: 34) [14].

The median VAS score in the symptomatic group was 5.0 (range 1–9) (Table 2). None of them used painkillers daily or before the endometrial sampling. These symptomatic women reported a median VAS score of 4.0 (range 1–8) in the 12 premenopausal, and 5.0 (range 1–9) in the 38 postmenopausal women. Two women were nulliparous and reported a median VAS score of 3.0 and in 33 multiparous women the median VAS score was 4.5 (range 1–8). Of 15 women the obstetrical history was unknown (median VAS score 5.0 (range 2–9).

## Discussion

Endometrial sampling, irrespective of indication, is a painful procedure, with a median VAS score of 5.0 in asymptomatic women with LS and in women with abnormal bleeding. We observed no progressive pain scores in subsequent procedures in the group women with LS. Of 52 women in the LS group, 7 (13 %) decided for preventive surgery and gave pain at the annual surveillance besides of fear for cancer, as an important reason for the preventive surgery. LS women reported no more pain during annual endometrial sampling than the symptomatic women without LS who underwent single endometrial sampling, although a substantial proportion of these LS women (11/52) used painkillers during subsequent endometrial samplings.

This is the first study that describes the level of pain during endometrial sampling in asymptomatic and symptomatic women and the influence of pain scores on clinical decision making during annual surveillance in women with LS. A limitation of this study is the small number of patients analysed because LS is not a very common trait.

In the last decades more LS families are detected and more annual gynaecologic surveillance procedures are performed in these women. In literature, the effectiveness of surveillance in women with LS and the role of preventive surgery have been described before. However, as far as we know, there is no information about the degree of pain as well as the influence of pain scores on clinical decision-making during surveillance in women with LS. Only one study reported on patient acceptability of endometrial sampling in LS surveillance and the authors conclude that transvaginal ultrasonography is associated with less discomfort than hysteroscopy or endometrial sampling, and will therefore be the preferred test of choice for the majority [15]. There is no significant difference between the pain scores for hysteroscopy and endometrial sampling [15]. In studies reporting on preventive surgery in women with LS no information is given so far about the influence of pain during the surveillance visits in making the decision for preventive surgery [16, 17]. Main outcome of this study was pain during the endometrial sampling, although there are a lot of factors that should be considered by physicians in making surveillance decisions with their patients. This includes, beside the level of pain and inconvenience, fear for cancer, having to come to the hospital, travel opportunities, the risks and benefits of surgery, child bearing wish and the costs of the surveillance versus preventive surgery. In this study we focussed on the pain because we had the impression that women who underwent repetitive annual endometrial sampling reported more pain than symptomatic non-LS women who got single endometrial sampling. Because women with LS undergo colon surveillance as well, we searched for literature about pain scores during surveillance for colon cancer. Nebgen et al. [18] described combined endometrial sampling and colonoscopy in 55 women with LS under conscious sedation and concluded that this combination of surveillance is a less painful experience in women with LS than endometrial sampling in an office setting without sedation. Huang et al. also described combined surveillance by endometrial sampling and colonoscopy under conscious sedation in 42 women with LS of whom 19 women had a previous endometrial sampling in the office setting. These women reported significantly lower pain levels in the combined procedure compared to the previous office procedure without sedation [19]. In this study 11/52 (21 %) LS women used painkillers (NSAID’s) before the endometrial sampling was performed, due to painful experiences during previous procedures. In the randomised controlled trial of Somchit et al. [20] women showed a significantly reduced pain score during the endometrial sampling in the group who used an NSAID compared to the group who used placebo. In our study, there were no statistically significant differences between postmenopausal and nulliparous women in the reported median VAS scores as compared to premenopausal and multiparous women. We expected a difference, as it might be that the endocervix is less easy to pass in postmenopausal and nulliparous women what might contribute to a higher level of pain during the procedure. In this study, 11 women used painkillers before the subsequent endometrial sampling because of a painful procedure during the last surveillance visit (VAS 7–10). These 11 women were almost all (8/11) premenopausal and only four of 11 women were nulliparous.

In this study nine women choose for preventive surgery, mostly because of fear for pain. All had normal findings and were a-symptomatic since the last surveillance, performed 10 (3–24) months before surgery. They did not undergo endometrial sampling shortly before the preventive surgery. Although there are no recommendations for endometrial sampling prior to risk reducing surgery in women with Lynch syndrome, some authors report on occult endometrial cancers in women with Lynch syndrome [1, 21] and others report only symptomatic endometrial cancers found by complaints and/or thick endometrial response on transvaginal ultrasound [12, 22]. In this study none of the nine pathology reports of the women who choose for preventive surgery showed a (pre)malignancy of the endometrial tissue. In conclusion, irrespective of the indication, women report substantial pain scores during repetitive and single endometrial sampling, although pain scores differ substantially between individuals. In this study it could not be confirmed that pain increases during subsequent endometrial samplings in women with LS. A substantial proportion (13 %) of these women decided for preventive surgery and gave pain at annual surveillance, besides fear for cancer, as a major reason for this decision. We suggest that more attention is needed for the impact of pain during endometrial sampling and use of painkillers should be encouraged (NSAID’s). Other less painful methods such as combining with colonoscopy under conscious sedation [18, 19], liquid biopsies in blood [23] and analysing endometrial tissue to detect endometrial cancer by tampons [24] should be explored to omit painful repetitive endometrial sampling in women with LS.
